# Vitamin B12 Status and Cardiovascular Risk: Novel Insights from NMR-Based Lipoprotein Profiling in 20,665 Adults

**DOI:** 10.3390/jcm15051775

**Published:** 2026-02-26

**Authors:** Yunus Eken, Nazlıhan Tekin, Furkan Şahin, İrem Tay, Neslihan Yıldırım Saral, Mustafa Serteser, Ahmet Tarık Baykal

**Affiliations:** 1 Department of Translational Medicine, Graduate School of Health Sciences, Acibadem Mehmet Ali Aydinlar University, Istanbul 34752, Türkiye; yunusekn5@gmail.com; 2 Acibadem Labmed Clinical Laboratories, Istanbul 34752, Türkiye; nazlihanyildirim@hotmail.com (N.T.); furkan.sahin1@live.acibadem.edu.tr (F.Ş.); irem.tay@live.acibadem.edu.tr (İ.T.); neslihan.saral@acibademlabmed.com.tr (N.Y.S.); mustafa.serteser@gmail.com (M.S.); 3 Department of Biochemistry and Molecular Biology, Graduate School of Health Sciences, Acibadem Mehmet Ali Aydinlar University, Istanbul 34752, Türkiye; 4 Department of Medical Biochemistry, School of Medicine, Acibadem Mehmet Ali Aydinlar University, Istanbul 34752, Türkiye

**Keywords:** vitamin b12, atherogenic index of plasma, NMR spectroscopy, lipoprotein subfractions, cardiovascular risk, VLDL, LDL

## Abstract

**Background/Objectives:** Vitamin B12 deficiency affects up to 40% of certain populations worldwide and has been associated with cardiometabolic risk. However, its relationship with detailed lipoprotein subfractions remains poorly defined. This study aimed to investigate the association between serum vitamin B12 levels and atherogenic lipid profiles using NMR-based lipoprotein subfraction analysis. **Methods:** In this retrospective cross-sectional study, data from 20,665 apparently healthy adults undergoing routine health screening were analyzed. Participants were categorized into quartiles based on serum vitamin B12 levels (Q1: ≤328 pg/mL; Q4: ≥540 pg/mL). Lipoprotein subfractions were quantified using Bruker NMR spectroscopy. The Atherogenic Index of Plasma was calculated as log_10_(triglycerides/HDL-cholesterol). Statistical analyses included ANCOVA adjusted for age, with corrections for multiple comparisons. **Results:** Higher serum B12 levels were significantly associated with a more favorable lipid profile. Specifically, AIP values decreased progressively across B12 quartiles (Q1: −0.051 ± 0.273; Q4: −0.170 ± 0.294; *p* < 0.001, partial η^2^ = 0.017). HDL-cholesterol increased (*p* < 0.001, partial η^2^ = 0.008), while triglycerides and VLDL-TG subfractions (VLDL1-TG: *p* < 0.001; VLDL5-TG: *p* = 0.029) declined with higher B12 levels. Among LDL subfractions, small dense LDL (LDL5-TG) exhibited a consistent inverse association with B12 (*p* = 0.002, partial η^2^ = 0.001). These associations were robust across all age strata, with no significant interaction between B12 levels and age. **Conclusions:** Serum vitamin B12 levels are inversely associated with atherogenic lipid parameters in a large cohort of asymptomatic individuals. Higher B12 status correlates with lower AIP, reduced triglyceride-rich lipoproteins, and diminished small dense LDL particles across all age groups. These findings suggest that B12 status may serve as a potential biomarker in cardiovascular risk assessment and highlight the need for prospective interventional studies.

## 1. Introduction

Vitamin B12 (cobalamin) is a water-soluble essential vitamin and a critical cofactor in fundamental biological processes, including erythropoiesis, nucleic acid metabolism, and the methylation cycle [1,2,3]. Through its role in regulating one-carbon metabolism via the enzymes methionine synthase and methylmalonyl-CoA mutase, B12 exerts a direct effect on cellular proliferation and gene expression [4]. This multifaceted mechanism of action explains why B12 deficiency disrupts systemic metabolic homeostasis, manifesting not only in classic hematological and neurological symptoms but also in broader metabolic dysregulation [3,5,6,7]. Vitamin B12 deficiency can develop through various pathways, such as malabsorption, inadequate nutrition, or genetic predisposition, and its clinical manifestations can range from mild biochemical abnormalities to severe neurological disorders [8,9,10]. The global burden of this deficiency is considerable, with prevalence rates reaching up to 40% in some populations, posing a serious public health problem, particularly among women of childbearing age and pregnant individuals [4,11,12,13]. This widespread deficiency has led to research into its broader metabolic consequences beyond traditional hematological and neurological symptoms. Accumulating evidence suggests that inadequate vitamin B12 levels may play a contributory role in the development of cardiometabolic disorders such as obesity, insulin resistance, and type 2 diabetes (T2DM) [14,15]. The underlying mechanisms of this relationship involve multiple interconnected pathways. Two primary mechanisms have been identified: first, B12 deficiency leads to elevated total homocysteine (tHcy) levels, which subsequently promotes the secretion of very low-density lipoprotein (VLDL); second, methylmalonyl-CoA accumulation suppresses fatty acid oxidation while simultaneously increasing lipogenesis [16,17,18,19,20,21,22]. These biochemical changes suggest that B12 may function as an important epigenetic and metabolic modulator in the development of atherogenic lipid profiles, thereby establishing a mechanistic link between B12 status and cardiovascular risk [23,24,25,26].

Cardiovascular disease (CVD), largely driven by atherosclerosis, remains the leading global cause of mortality [19,27]. Atherosclerosis involves a complex interplay of lipid deposition, endothelial dysfunction, and chronic inflammation. B12 deficiency exacerbates these processes through hyperhomocysteinemia, which increases oxidative stress, reduces nitric oxide availability, induces endothelial apoptosis, and fosters a prothrombotic state [20,28,29]. Notably, experimental models have demonstrated that B12 deficiency can promote atherogenesis independently of hypercholesterolemia [4,30,31], implicating direct pathogenic mechanisms beyond homocysteine. Epidemiological data further support this relationship, with each 5 μmol/L increase in serum homocysteine above 10 μmol/L associated with a 20% rise in cardiovascular event risk [32]. These findings highlight the need to assess extended lipid parameters beyond conventional markers in cardiovascular risk evaluation. In cardiovascular risk assessment, traditional lipid parameters (LDL-cholesterol, HDL-cholesterol, total cholesterol) alone may be insufficient to capture the full spectrum of atherogenic risk [33,34]. In this context, the Atherogenic Index of Plasma (AIP), which reflects the balance between triglycerides (TG) and HDL-cholesterol, has emerged as a composite indicator that better represents the dynamic biology of atherosclerosis. Calculated using the formula log_10_(TG/HDL-C), AIP shows strong correlations with low-density lipoprotein (LDL) particle size, cholesterol esterification rate, and small dense LDL (sdLDL) formation [35,36,37]. Prospective studies have shown that AIP independently predicts cardiovascular events beyond traditional risk factors and can detect increased risk even when standard lipid values are within the normal range [38,39,40]. Given the potential effects of vitamin B12 on both triglyceride metabolism and high-density lipoprotein (HDL) levels, the relationship between this vitamin and AIP represents a critical area of research for cardiovascular risk assessment. Despite growing interest in the metabolic effects of B12, the current literature presents heterogeneous findings regarding the relationship between B12 status and lipid profiles; most studies focus on populations with existing comorbidities [14,41]. The effect of B12 on high-resolution lipoprotein subfractions, particularly VLDL and LDL subclasses, in apparently healthy individuals has not been extensively investigated [1,2,41,42]. Furthermore, studies evaluating the relationship between B12 and AIP in large population samples are quite limited, and potential age-related variations in these relationships have not been systematically examined. This study aims to comprehensively investigate the relationship between serum vitamin B12 concentrations, divided into four quartiles, and markers reflecting the atherogenic lipid phenotype in a large and apparently healthy population [27,43]. In addition to classic lipid parameters and AIP [33,44], we evaluated VLDL-TG subfractions and selected LDL-TG subfractions using nuclear magnetic resonance (NMR) spectroscopy. Furthermore, to address a gap in the existing literature, we created age groups to investigate whether these relationships differ by age. Our primary hypothesis is that parameters reflecting triglyceride-rich lipoprotein load, particularly VLDL-TG subfractions, will exhibit a more adverse pattern in lower B12 quartiles, and that this relationship may vary across age groups. These findings may provide insights into the role of B12 status in cardiovascular risk classification and shed light on targeted intervention strategies.

## 2. Materials and Methods

### 2.1. Study Design

This retrospective cross-sectional observational study analyzed routine clinical and laboratory data from individuals without known/diagnosed diseases who applied for check-ups at Acıbadem Health Group hospitals between 2023–2025. Individuals with available serum vitamin B12 measurements and NMR-based lipoprotein subfraction outputs were included. All participants fasted for at least 8–12 h before blood collection. Participants were evaluated in the 18–≥75 age range and were divided into predefined age groups (18–44, 45–59, 60–74, and ≥75 years).

### 2.2. Ethical Approval

This study utilized previously collected, anonymized metabolic datasets. Written informed consent had been obtained from all participants for the use of these datasets. Ethical approval for the use of the dataset was granted by the Acıbadem Mehmet Ali Aydınlar University Medical Research Evaluation Board (ATADEK) on 11 September 2025 (Decision No: 2025-13/49). All procedures were conducted in accordance with the Declaration of Helsinki and applicable local regulations.

### 2.3. Vitamin B12 Measurement

Serum vitamin B12 levels were quantitatively measured using a direct chemiluminescence-based competitive immunoassay method with the Atellica IM Vitamin B12 (VB12) kit on the Atellica IM Analyzer (Siemens Healthineers, Erlangen, Germany). Sample B12 competes with acridinium ester-labeled B12 for intrinsic factor bound to paramagnetic particles; the signal-to-luminosity (RLU) is inversely proportional to B12 concentration. 100 µL of sample was used for each measurement; the measurement range was accepted as 45–2000 pg/mL. According to the manufacturer’s guidelines and clinical practice standards, serum B12 levels of 211–911 pg/mL were defined as normal, while levels < 211 pg/mL were classified as B12 deficiency. Notably, participants with serum B12 levels below 211 pg/mL (i.e., those meeting the laboratory criterion for B12 deficiency) were included in the study and were categorized within the first quartile (Q1: ≤328 pg/mL). These individuals were not excluded from the primary analyses, as the study aimed to capture the full spectrum of B12 status in the population. A sensitivity analysis excluding participants with B12 levels < 211 pg/mL is recommended in future investigations to assess potential confounding by overt B12 deficiency. All measurements were performed in the Acıbadem Labmed Clinical Laboratories.

### 2.4. Nuclear Magnetic Resonance Analysis

Lipoprotein profiling was performed using the Bruker IVDR NMR system (Bruker BioSpin, Rheinstetten, Germany). 400 µL of serum sample was mixed with 400 µL of buffer containing 20% D_2_O, 0.075 M sodium monophosphate, 4.6 mM 3-(trimethylsilyl) propionic-2,2,3,3-d4 acid (TSP), and 0.04% NaN_3_, and transferred to 5 mm NMR tubes. Lipid and lipoprotein subclass profiles were automatically determined (very-low-density lipoprotein [VLDL], intermediate-density lipoprotein [IDL], low-density lipoprotein [LDL], high-density lipoprotein [HDL] subfractions) using the Bruker IVDr Lipoprotein Subclass Analysis (B.I.LISA™) platform. For frozen serum samples, samples were thawed at +4 °C, gently rotated for 4 min, and processed following the same preparation protocol to ensure consistency between fresh and frozen samples.

### 2.5. NMR Data Acquisition

Spectra were acquired at 310 K using a water-suppressed NOESY (noesygppr1d) array on a Bruker Avance Neo 600 MHz IVDr spectrometer (Bruker, Billerica, MA, USA) equipped with a 5 mm BBI probe, SampleJet, TopSpin v4.5 software. Acquisition parameters included: 32 scans, 30 ppm spectral width, 4 dummy scans, 4 s relaxation delay, and a total acquisition time of 4:04 min.

### 2.6. Derived Variables and Data Transformation

Participants were divided into quartiles according to serum B12 levels: (Q1: ≤328 pg/mL, Q2: 329–417 pg/mL, Q3: 418–539 pg/mL, Q4: ≥540 pg/mL). These cutoffs were determined based on the distribution of B12 values in the study population to ensure approximately equal sample sizes across quartiles, thereby maximizing statistical power for between-group comparisons. The quartile-based approach was chosen over clinical deficiency thresholds because the study aimed to evaluate the association between B12 status across the full population distribution rather than comparing only deficient versus non-deficient individuals. This approach allows detection of potential dose–response relationships across the entire B12 spectrum, including within the reference range. The Atherogenic Index of Plasma (AIP) was calculated as log10(TG/HDL-C). Serum vitamin B12 and selected lipoprotein subfraction variables (especially VLDL-TG and LDL-TG subgroups) with a right-skewed distribution were subjected to ln(x + 1) transformation to approximate normality and were used in the analysis on the ln-scale. Variables that met normality assumptions without transformation (AIP, HDL-C, LDL-C, total cholesterol) were analyzed on their original scale. All figures and tables indicate whether values represent transformed or untransformed data.

### 2.7. Statistical Analysis

Statistical analyses were performed using IBM SPSS Statistics for Windows, version 26.0 (IBM Corp., Armonk, NY, USA); visualizations were created using GraphPad Prism for Windows, version 8.0 (GraphPad Software, San Diego, CA, USA). Continuous variables were presented as mean ± standard deviation (SD), and categorical variables as frequencies and percentages. One-way ANOVA was used to compare continuous variables across vitamin B12 quartiles, followed by post hoc Tukey HSD and Bonferroni corrections for multiple comparisons. Chi-square (χ^2^) test was used for categorical variables. Two-way ANOVA and General Linear Model (GLM) with ANCOVA were employed to assess the effects of B12 quartiles, age groups, and sex, as well as their interactions, on lipid parameters. Effect sizes were reported as partial η^2^. To control for the potential confounding effects of age on both Vitamin B12 status and lipid metabolism, all comparative analyses were adjusted for age groups. Unless otherwise noted, observed associations and statistical significance levels were consistent across all age categories; therefore, combined data or general trends are presented to avoid repetition in the text. Statistical significance was set at *p* < 0.05 for all analyses.

## 3. Results

### 3.1. Baseline Characteristics of the Study Population

A total of 20,665 individuals were included in the study, and participants were divided into four groups based on their serum vitamin B12 levels (Q1: ≤328 pg/mL, Q2: 329–417 pg/mL, Q3: 418–539 pg/mL, Q4: ≥540 pg/mL). The demographic and biochemical characteristics of participants according to B12 quartiles are summarized in Table 1. Age and sex distribution differed significantly between B12 quartiles (*p* < 0.001 for both). Within the entire sample group, women had higher B12 levels than men (Figure S1). As vitamin B12 levels increased, the proportion of women in the group rose from 41.8% (Q1) to 49.6% (Q4), and the mean age showed a slight upward trend (*p* < 0.001). In lipid profiles, the highest vitamin B12 group showed a statistically significant decrease in TG levels (from 132.01 mg/dL to 108.58 mg/dL) and total VLDL-TG levels (from 84.13 mg/dL to 65.55 mg/dL) compared to the lowest group (*p* < 0.001). Parallel to the increase in serum B12 concentration, a significant increase in HDL-cholesterol levels and a significant improvement in AIP values were observed (*p* < 0.001). Although a slight increase in total LDL-cholesterol and total cholesterol levels was observed with the increase in B12 (*p* < 0.001), a significant decrease in total LDL-TG levels (*p* = 0.014) was recorded.

### 3.2. Vitamin B12 and Atherogenic Index of Plasma (AIP)

**Figure 1:**
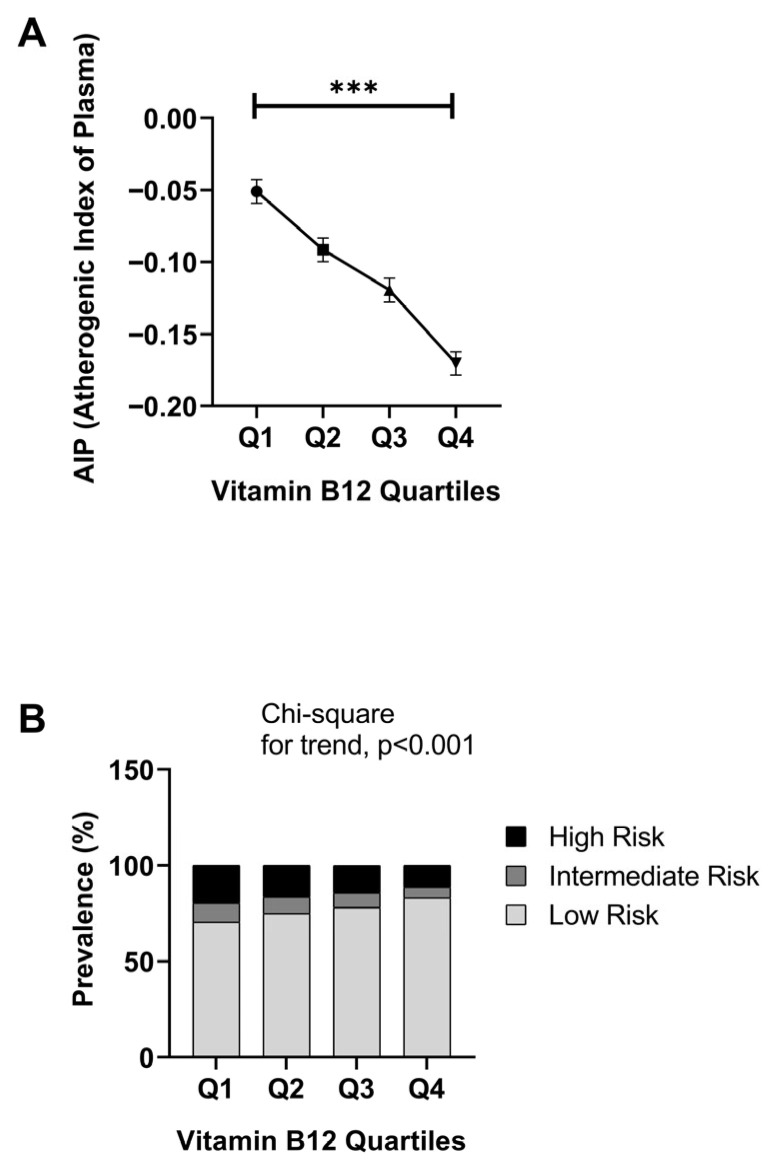
Distribution of Atherogenic Index of Plasma and AIP risk categories according to Vitamin B12 quartiles: (**A**) Mean AIP values across Vitamin B12 quartiles. AIP was calculated as log10(TG/HDL-C). Interquartile differences were evaluated by one-way ANOVA (F(320,661) = 143.977; *p* < 0.001; partial η^2^ = 0.020), and multiple comparisons were confirmed by Tukey HSD (*p* < 0.001 for all). Data are presented as means with 95% confidence intervals (CI). Error bars represent 95% CI. In the graph, *** indicates *p* < 0.001 for one-way ANOVA. The line with brackets indicates significant difference between groups. (**B**) Prevalence of risk categories according to AIP across B12 quartiles. Risk classification thresholds: low risk (AIP < 0.11), moderate risk (0.11–0.24) and high risk (AIP > 0.24). Linear trends in categorical distribution across quartiles were assessed using the Chi-square trend test (*p* < 0.001). Vitamin B12 quartile ranges: Q1 ≤328 pg/mL; Q2 329–417 pg/mL; Q3 418–539 pg/mL; Q4 ≥540 pg/mL.

Across the vitamin B12 quartiles, highly significant differences were found in AIP scores among all B12 groups using one-way ANOVA analysis, which is used as an indicator of atherogenic risk to assess the cardiovascular risk profile (ANOVA: F(3, 20,661) = 143.977; *p* < 0.001; partial η^2^ = 0.020). As seen in Figure 1A, a regular and significant decrease in AIP values was observed as vitamin B12 levels increased. The highest AIP values were found in the lowest B12 group (≤328 pg/mL; Mean = −0.051 ± 0.30), while the lowest AIP values were recorded in the highest B12 group (≥540+ pg/mL; Mean = −0.170 ± 0.29). In multiple comparisons, pairwise differences between all B12 quartiles were confirmed to be statistically significant after both Tukey HSD and Bonferroni correction (*p* < 0.001 for all comparisons). When risk stratification was performed according to AIP thresholds (low risk: AIP < 0.11; moderate risk: 0.11–0.24; high risk: >0.24), the distribution of risk categories showed a significant linear trend across the B12 quartiles; the proportion of low-risk individuals increased in higher B12 quartiles while the proportion of high-risk individuals decreased (Chi-square trend test: *p* < 0.001; Figure 1B).

A two-way ANOVA analysis was performed to evaluate the effects and potential interactions of B12 quartiles and sex on AIP (Table 2). Both B12 quartile (F = 120.589; *p* < 0.001; partial η^2^ = 0.017) and sex (F = 3423.521; *p* < 0.001; partial η^2^ = 0.142) showed significant main effects on AIP. Notably, the B12 × sex interaction was found to be statistically significant (F = 5.345; *p* = 0.001; partial η^2^ = 0.001), indicating that the relationship between B12 status and AIP differed between men and women. The effect size of sex (partial η^2^ = 0.142) was significantly higher than the effect size of the B12 quartiles (partial η^2^ = 0.017), suggesting that sex is the dominant factor determining AIP in this population. Men consistently exhibited higher AIP values compared to women across all B12 quartiles (men: −0.005 ± 0.298 vs. women: −0.237 ± 0.258; *p* < 0.001). In the lowest B12 quartile, men reached positive AIP values (0.045 ± 0.295) and approached the intermediate cardiovascular risk threshold; in contrast, women maintained negative values across all quartiles.

### 3.3. Vitamin B12 and Classical Lipid Parameters (HDL-C and LDL-C)

**Figure 2:**
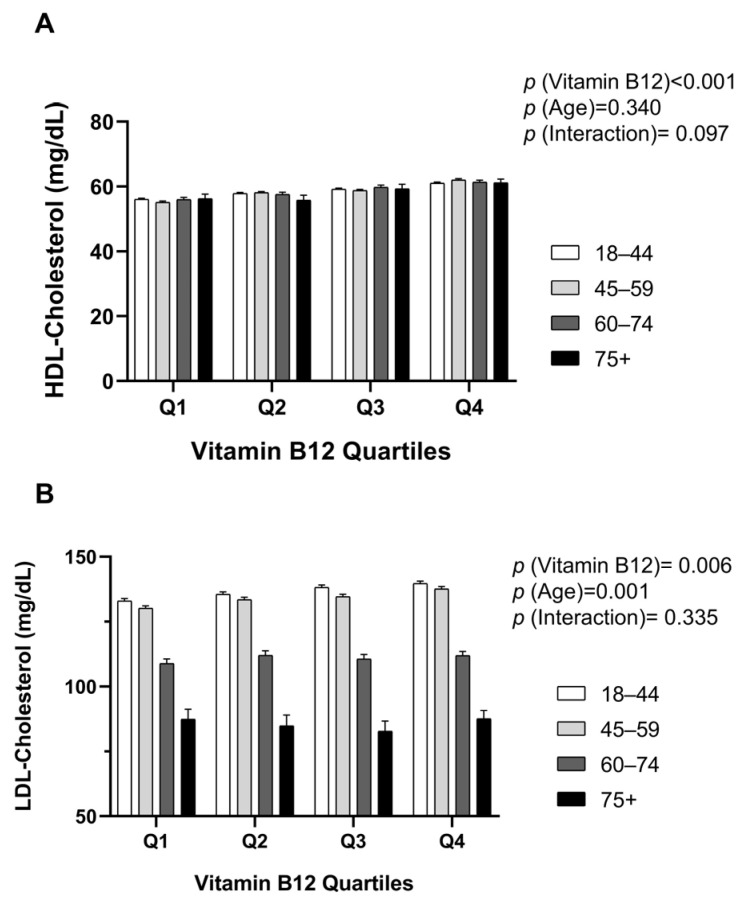
Classical lipid parameters across vitamin B12 quartiles stratified by age groups: (**A**) High-density lipoprotein cholesterol (HDL-C) levels across four vitamin B12 quartiles in four age groups. B12 quartiles: Q1 (≤328 pg/mL, *n* = 5175), Q2 (329–417 pg/mL, *n* = 5160), Q3 (418–539 pg/mL, *n* = 5152), Q4 (≥540 pg/mL, *n* = 5178). Age groups: 18–44 years (blue circles), 45–59 years (red squares), 60–74 years (green triangles), ≥75 years (purple diamonds). Data are presented as estimated marginal means ± 95% confidence intervals (CI) from analysis of covariance (ANCOVA) with age as a continuous covariate. Main effect of B12 quartile: F = 56.321, *p* < 0.001, partial η^2^ = 0.008; main effect of age group: F = 0.916, *p* = 0.340, partial η^2^ < 0.001; B12 × age group interaction: F = 1.893, *p* = 0.097, partial η^2^ = 0.001. The overall model R^2^ = 0.022 (adjusted R^2^ = 0.022). (**B**) Low-density lipoprotein cholesterol (LDL-C) levels across B12 quartiles and age groups. Data presentation and symbols as in panel A. Main effect of B12 quartile: F = 3.787, *p* = 0.006, partial η^2^ = 0.001; main effect of age as covariate: F = 364.186, *p* < 0.001, partial η^2^ = 0.017; main effect of age group: F = 154.132, *p* < 0.001, partial η^2^ = 0.033; B12 × age group interaction: F = 1.141, *p* = 0.335, partial η^2^ < 0.001. Overall model R^2^ = 0.044 (adjusted R^2^ = 0.043).

The relationship between vitamin B12 quartiles and classical lipid parameters (HDL-C and LDL-C) is shown in Figure 2, stratified by age groups. ANCOVA analysis for HDL-C levels revealed a significant main effect of vitamin B12 quartiles (F = 56.321, *p* < 0.001, partial η^2^ = 0.008). Total cholesterol levels showed a similar pattern across B12 categories and age groups, with higher B12 status associated with more favorable cholesterol distribution, particularly in the 45–59 and 60–74 age groups (Figure S2). HDL-C levels progressively increased from Q1 (55.2 ± 0.2 mg/dL) to Q4 (61.5 ± 0.2 mg/dL). The main effect of age groups was not statistically significant (F = 0.916, *p* = 0.340, partial η^2^ < 0.001), and the B12 × age group interaction was also not significant (F = 1.893, *p* = 0.097, partial η^2^ = 0.001). These findings demonstrate that the positive association between B12 and HDL-C is consistent across all age groups (Figure 2A).

ANCOVA analysis of LDL-C levels revealed a statistically significant but modest main effect of vitamin B12 quartiles (F = 3.787, *p* = 0.006, partial η^2^ = 0.001). A slight increase in LDL-C levels was observed in higher B12 quartiles, though the effect size was very small (partial η^2^ = 0.001), suggesting limited clinical relevance for this parameter. However, a strong main effect of age was detected (F = 364.186, *p* < 0.001, partial η^2^ = 0.017). Specifically, LDL-C levels were significantly lower in the 75 years and older group (~88.5 ± 1.1 mg/dL) compared to the 18–44 years group (~132.5 ± 0.8 mg/dL). The B12 × age group interaction was not statistically significant (F = 1.141, *p* = 0.335, partial η^2^ < 0.001; Figure 2B).

### 3.4. VLDL-Triglyceride Subfractions Across B12 Quartiles

**Figure 3:**
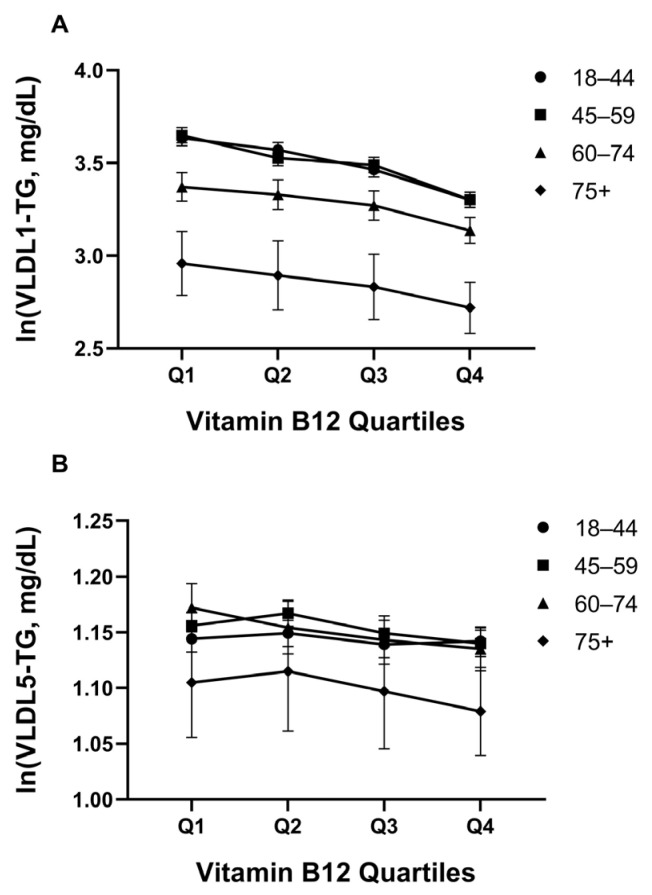
Changes in VLDL-TG subclass triglyceride concentrations according to vitamin B12 quartiles. (**A**) Relationship between vitamin B12 groups and VLDL-1 TG levels. A linear decrease in VLDL-1 TG values is observed in parallel with increasing serum B12 concentration (*p* < 0.001, ANOVA). (**B**) Relationship between vitamin B12 groups and VLDL-5 TG levels. TG content in the VLDL-5 fraction was found to be significantly lower in individuals with high B12 levels (*p* < 0.001, ANOVA). Data are expressed as mean with 95% confidence intervals (CI). Error bars represent 95% CI. Q1: ≤328 pg/mL; Q2: 329–417 pg/mL; Q3: 418–539 pg/mL; Q4: ≥540 pg/mL.

Vitamin B12 quartiles and triglyceride-rich lipoprotein load were assessed via ln(x + 1) converted VLDL-TG subfractions. In GLM/ANCOVA analysis, the main effect of B12 for VLDL1-TG was significant (*p* < 0.001; partial η^2^ = 0.005; R^2^ = 0.047), and VLDL1-TG decreased overall as B12 quartiles increased; age and age group also contributed significantly (*p* < 0.001 for both), but the B12 × age group interaction was not significant (*p* = 0.380) (Figure 3A). The main effect of B12 for VLDL5-TG was smaller but significant (*p* = 0.029; R^2^ = 0.084), and again no interaction was detected (*p* = 0.424, Figure 3B); However, no significant difference was maintained in pairwise comparisons after Bonferroni analysis (all *p* ≥ 0.071). Age-stratified analysis of the medium and smaller VLDL-TG subfractions (VLDL2-TG, VLDL3-TG, and VLDL4-TG) revealed consistent inverse associations with B12 quartiles across all age groups (Figure S3). While baseline VLDL-TG levels increased with age (particularly in the 45–59 and 60–74 age groups), the protective association of higher B12 status with lower triglyceride load was maintained throughout life. The most pronounced absolute differences between B12 quartiles were observed in middle-aged adults (45–74 years), suggesting that adequate B12 status may be particularly important for cardiovascular risk management in this age range. Notably, even in the oldest age group (75+ years), higher B12 quartiles were associated with lower VLDL-TG levels, indicating that the metabolic benefits of optimal B12 status extend into advanced age.

Effect size analysis revealed a systematic pattern in the strength of associations between B12 and VLDL-TG subfractions (Figure S4). The largest effect was observed for VLDL1-TG (partial η^2^ = 0.016, *p* < 0.001), representing the largest and most triglyceride-rich particles, followed by progressively smaller effects for VLDL2-TG (η^2^ = 0.009), VLDL3-TG (η^2^ = 0.010), VLDL4-TG (η^2^ = 0.006), and VLDL5-TG (η^2^ = 0.001). This decreasing effect size trend suggests that the association between vitamin B12 and VLDL-TG is most pronounced for hepatically secreted, larger VLDL particles, potentially reflecting differences in hepatic triglyceride synthesis and VLDL assembly rather than in smaller remnant particles generated through lipolysis.

### 3.5. LDL Triglyceride Subfractions Across B12 Quartiles

**Figure 4:**
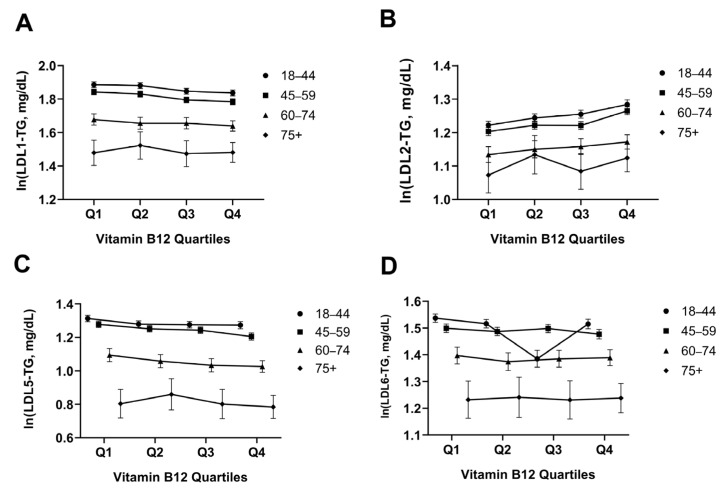
Changes in LDL-TG subfractions across age groups throughout Vitamin B12 quartiles. (**A**) LDL1-TG, (**B**) LDL2-TG, (**C**) LDL-5-TG and (**D**) LDL-6-TG values are shown in age groups according to Vitamin B12 quartiles. Symbols represent the estimated marginal means of log-transformed [ln(x + 1)] values with 95% confidence intervals (CI) (model covariate: age). Error bars represent 95% CI. Statistical analysis was performed using GLM/ANCOVA; reported *p*-values represent the main effect of the B12 quartile and the B12 × age group interaction, effect size is given as partial eta-squared (partial η^2^). Multiple comparisons were performed with Bonferroni correction. Q1: ≤328 pg/mL; Q2: 329–417 pg/mL; Q3: 418–539 pg/mL; Q4: ≥540 pg/mL.

The relationship between vitamin B12 quartiles and triglyceride subfractions was evaluated using a stratified GLM/ANCOVA model with age as a covariate (Figure 4). B12 quartiles were associated with LDL1-TG (*p* = 0.005; partial η^2^ = 0.001, Figure 4A) and LDL2-TG levels (*p* < 0.001; partial η^2^ = 0.002, Figure 4B), and no B12 × age group interaction was observed in these effects (*p* = 0.843 and *p* = 0.490, respectively). In LDL subfractions, B12 quartiles showed a significant main effect for LDL-5-TG (*p* = 0.002; partial η^2^ = 0.001, Figure 4C), but not for LDL-6-TG (*p* = 0.774, Figure 4C). The interaction was not significant in either parameter (*p* = 0.457 and *p* = 0.910). Age/age group variables made a significant contribution in all models (*p* < 0.001).

### 3.6. Age-Stratified Analysis of Total Triglycerides

**Figure 5:**
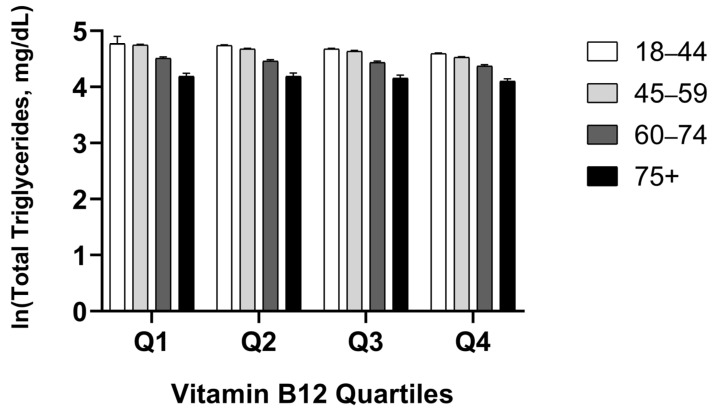
Total triglyceride levels across vitamin B12 quartiles, classified by age groups. Data are presented as means with 95% confidence intervals (CI) for each age group. Error bars represent 95% CI. Two-way ANOVA: Main effect of B12 quartile (F = 28.733; *p* < 0.001), main effect of age group (F = 105.216; *p* < 0.001), B12 × age group interaction (F = 1.448; *p* = 0.161). The insignificant interaction indicates that the inverse relationship between B12 and triglycerides is consistent across all age groups. White: 18–44 years; light grey: 45–59 years; dark grey: 60–74 years; black: 75+ years. Q1: ≤328 pg/mL; Q2: 329–417 pg/mL; Q3: 418–539 pg/mL; Q4: ≥540 pg/mL.

A two-way ANOVA analysis was performed to evaluate the effects of B12 quartiles and age groups on ln-transformed total triglyceride levels (Figure 5). Both B12 quartile (F = 28.733; *p* < 0.001) and age group (F = 105.216; *p* < 0.001) showed significant main effects on triglyceride levels. The model explained 7.2% of the variance in triglyceride levels (R^2^ = 0.072). Importantly, the B12 × age group interaction was not statistically significant (F = 1.448; *p* = 0.161), indicating that the inverse relationship between B12 status and triglyceride levels was consistent across all age groups. Age-adjusted B12 quartile estimated marginal means showed a gradual decrease: Q1 (4.554; 95% CI: 4.524–4.585), Q2 (4.516; 95% CI: 4.483–4.549), Q3 (4.476; 95% CI: 4.444–4.507), and Q4 (4.400; 95% CI: 4.373–4.426). Age group analysis revealed that triglyceride levels decreased with age: 18–44 years (4.695; 95% CI: 4.678–4.712), 45–59 years (4.648; 95% CI: 4.634–4.661), 60–74 years (4.445; 95% CI: 4.413–4.477), and 75+ years (4.159; 95% CI: 4.096–4.222). A consistent pattern of decreasing triglycerides across B12 quartiles was observed in all age groups, with the 18–44 age group showing the highest values (Q1: 4.770 to Q4: 4.595) and the 75+ age group showing the lowest values (Q1: 4.189 to Q4: 4.101).

## 4. Discussion

This study is one of the largest cross-sectional studies in the literature to comprehensively evaluate the relationship between serum vitamin B12 levels and atherogenic lipid profile in a large population (*n* = 20,665). Our findings reveal that high serum B12 concentrations are consistently associated with a lower AIP, reduced triglyceride load, improved HDL-cholesterol levels, and lower VLDL-TG fractions. These associations were generally maintained across age groups, providing significant evidence for the potential role of B12 in cardiovascular risk modulation. Before examining these associations in detail, it is important to acknowledge that with a sample size exceeding 20,000 participants, even small differences can achieve statistical significance. The effect sizes in this study are generally modest (partial η^2^ values mostly ≤0.02), which, according to Cohen’s classification, correspond to small effects. Therefore, while the observed associations are statistically robust, their direct clinical relevance warrants cautious interpretation. Nevertheless, for parameters such as AIP, triglycerides, and VLDL-TG, the consistent dose–response pattern across B12 quartiles and the convergence with experimental evidence suggest biological plausibility beyond mere statistical artifact [45]. The most striking finding of our study is the gradual and significant decrease in AIP values observed across vitamin B12 quartiles. The mean AIP value was −0.051 in the lowest B12 group, while it decreased to −0.170 in the highest group.

AIP is a cardiovascular risk marker developed by Dobiášová and Frohlich and calculated using the formula log_10_(TG/HDL-C) [36,46]. In the literature, AIP < 0.11 is classified as low risk, 0.11–0.24 as moderate risk, and >0.24 as high risk [40,46,47]. As B12 quartiles increased, the proportion of low-risk individuals increased while the proportion of high-risk individuals decreased. Combined with the finding by Kim et al. that AIP independently predicted cardiovascular events beyond traditional risk factors, these results indicate that B12 status may play a role in cardiovascular risk stratification [39]. A study conducted in Iran reported that AIP showed strong correlations with other cardiovascular risk factors and that its use in risk assessment may be useful, especially when there are no abnormalities in standard lipid values [40]. Our findings support a potential association between B12 status and AIP. In particular, the fact that all pairwise comparisons between B12 quartiles retained their significance after both Tukey HSD and Bonferroni correction highlights the strength of these relationships. Similarly, a study of approximately 7000 people also showed that AIP can be a strong predictor of cardiovascular disease risk [46,48,49].

In our study, a significant decrease in triglyceride levels was observed across B12 quartiles, and this trend was even more pronounced for VLDL-TG. These findings appear consistent with clinical data showing that B12 deficiency is associated with a more unfavorable lipid profile. In the UK and India, B12 deficiency has been reported to be independently associated with high triglyceride levels in patients with T2DM [14]. Similarly, Mahalle et al., in a study conducted in India on 300 coronary artery disease patients, found that serum B12 showed an inverse correlation with triglycerides and VLDL, and a positive correlation with HDL [41]. Triglycerides, total cholesterol, and LDL cholesterol, which are known to be inversely related to serum B12 levels [1], showed differences in our study. In contrast to this information, a slight increase in total LDL-cholesterol levels was observed with increasing B12 levels in our study. This difference may be due to the populations included in previous studies or the size of the sample sizes [50,51].

In addition to clinical studies, experimental studies also provide mechanistic explanations for the effects of B12 on triglyceride metabolism. A study in the HepG2 hepatocyte cell line showed that low B12 levels increased lipogenesis, impaired fatty acid oxidation, and induced intracellular triglyceride accumulation [23]. The elevated triglyceride levels and increased VLDL-TG fractions observed in the lowest B12 quartile (Q1, ≤328 pg/mL, mean 268.71 ± 44.44 pg/mL) are consistent with enhanced hepatic lipogenesis demonstrated in cellular models. The gradual decrease in triglyceride values as B12 levels increase is consistent with a dose-dependent association between B12 status and hepatic de novo lipogenesis. Similarly, studies in experimental animals have shown that severe B12 deficiency significantly increases plasma triglyceride and cholesterol levels and decreases HDL levels [31]. Interestingly, the B12 threshold values in this animal model are comparable to the human quartile values in our study; the mean B12 level in our lowest quartile (268.71 ± 44.44 pg/mL) is approximately half that of control mice. Furthermore, the increase in HDL-cholesterol and decrease in triglycerides from Q1 to Q4 in our study parallel the lipid profile changes observed by Kumar et al. in their mouse model. Another animal study reported that maternal B12 deficiency in Wistar rats resulted in increased adiposity, high triglyceride and cholesterol levels in the offspring [31,52]. The significant difference in VLDL1-TG levels between B12 quartiles in our study may represent the reflection of these experimental findings in the human population. Specifically, the fact that VLDL1 particles are the largest triglyceride-rich VLDL subfraction and are considered a direct indicator of hepatic lipogenesis may support an association between B12 status and hepatic triglyceride production. This experimental evidence elucidates the biological mechanisms of the epidemiological observations in our study and provides a strong basis for the causal dimension of the B12-lipid relationship.

The gradual increase in HDL-C levels supports the potential role of B12 in cardiovascular protection. The cardiovascular protective effect of HDL occurs via reverse cholesterol transport. The inverse relationship between HDL and cardiovascular risk, first identified in the Framingham Heart Study, has demonstrated the importance of the RCT pathway [21,53,54]. Observational studies have shown that each 1 mg/dL increase in HDL-C reduces cardiovascular risk by approximately 2–3% [55]. The positive association between B12 and HDL-C observed in our study suggests that B12 status may be favorably associated with this critical antiatherogenic pathway.

Regarding LDL subfractions, the B12-related decrease in LDL5-TG (small dense LDL triglyceride) levels is particularly important, as sdLDL particles constitute the most atherogenic LDL subfraction [22,56,57,58]. The different effect sizes observed in VLDL subfractions provide mechanistic insights into how vitamin B12 affects triglyceride metabolism. The strongest association with VLDL1-TG, representing newly formed particles secreted in the liver, suggests that B12 may primarily be associated with hepatic triglyceride synthesis and VLDL formation rather than extrahepatic lipolytic processes. This is consistent with experimental evidence showing that B12 deficiency leads to the accumulation of methylmalonic acid, which can inhibit fatty acid β-oxidation and promote hepatic lipogenesis. Age-stratified analysis shows that although aging is associated with higher basal VLDL-TG levels, the protective effect of adequate B12 status persists across all age groups, supporting consideration of B12 assessment in cardiovascular risk evaluation regardless of age [59]. A study analyzing Women’s Health Study data reported that sdLDL cholesterol is associated with myocardial infarction risk independently of LDL-C and CRP [10]. In our study, the consistent observation of the B12-related decrease in LDL5-TG across all age groups (18–44, 45–59, 60–74, 75+) and the lack of a significant B12 × age group interaction suggest that this relationship is independent of age and has a strong biological basis. The significant decrease in LDL5-TG in Q3-Q4, particularly in the 75+ age group, suggests that adequate B12 levels in older adults may be important in improving the atherogenic LDL profile. In contrast, the irregular pattern in LDL6-TG (the smallest dense LDL) levels suggests that this fraction is more influenced by other metabolic determinants such as insulin resistance, visceral adiposity, or genetic factors rather than B12; indeed, Bernei and Krauss’s studies on the atherogenic lipoprotein phenotype emphasized that the formation of the smallest LDL particles is closely related to CETP (cholesterol ester transfer protein) activity and hepatic lipase expression. B12-related changes in LDL1-TG and LDL2-TG levels point to potential effects on lipoprotein lipase activity and peripheral triglyceride clearance. As defined in studies on the atherogenic lipoprotein phenotype (ALP), increased triglyceride transfer to LDL particles via CETP in a high triglyceride environment, followed by the conversion of these particles into smaller, denser forms by hepatic lipase, increases the risk of atherosclerosis [56]. Our findings suggest that B12 status may be associated with this atherogenic cascade [14,30,56]. The slight increase in total LDL-C appears paradoxical; however, this finding should be interpreted with caution, given its very small effect size (partial η^2^ = 0.001). The concurrent low triglyceride and high HDL profile in the high B12 groups may reflect a shift toward larger, more buoyant, and less atherogenic LDL particles [15,59,60,61]. This interpretation is consistent with the concept of the atherogenic lipoprotein phenotype, wherein a high-triglyceride environment promotes the formation of small dense LDL through CETP-mediated lipid exchange and hepatic lipase activity. In higher B12 quartiles, the reduced triglyceride load may favor the retention of larger LDL particles, which are considered less atherogenic [35,62,63]. Nevertheless, this explanation remains speculative in the absence of direct LDL particle size measurements, and future studies with comprehensive LDL subfraction characterization are needed to confirm this hypothesis.

In our study, a significant difference was found in the age distribution among the B12 quartiles; the mean age was 45.77 years in the lowest B12 group, while it increased to 47.37 years in the highest group. This finding differs from studies in the literature reporting decreased B12 absorption and increased deficiency prevalence with aging [64]. However, this suggests that individuals in the high B12 group may be using more B12 supplementation or that the study population consisted of healthy individuals. Additionally, the possibility that individuals in higher B12 quartiles may have been using B12 supplements cannot be excluded. Exogenous B12 supplementation could elevate serum B12 levels without necessarily reflecting improved intracellular B12 function, potentially confounding the observed associations. Unfortunately, supplementation data were not available in the current dataset, and this represents an important limitation. On the other hand, the clinically marginal age difference (approximately 1.6 years) and the lack of significant B12 × age group interactions for lipid parameters support the idea that the observed B12-lipid relationships are independent of age. In terms of sex distribution, a significant difference was observed between B12 quartiles [60]. The proportion of women increased from 41.8% in Q1 to 49.6% in Q4; the proportion of men decreased from 58.2% in Q1 to 50.4% in Q4. This finding indicates that men are more heavily represented in the low B12 groups. Literature reports suggest that men may be more susceptible to B12 deficiency compared to women due to higher alcohol consumption, smoking, and different dietary patterns [65]. Additionally, the higher likelihood of women using B12 supplements during pregnancy and childbearing periods may contribute to this difference [4]. Beyond the distributional differences, the sex-stratified analyses revealed significant B12 × sex interactions for several lipid parameters, suggesting that the metabolic consequences of low B12 status may not be uniform across sexes. This significant B12 × sex interaction may be explained by several biological mechanisms. First, estrogen is known to enhance hepatic LDL receptor expression and promote favorable HDL metabolism, which may buffer the effects of low B12 on atherogenic lipid profiles in women [54,66,67]. Second, sex-specific differences in visceral adiposity, hepatic lipase activity, and CETP function may modulate the relationship between B12 and triglyceride-rich lipoprotein metabolism differently in men and women [12,45,55,67]. Third, the higher prevalence of metabolic risk factors (e.g., visceral obesity, insulin resistance) in men may amplify the adverse lipid effects of lower B12 status. These sex-dependent differences have important clinical implications and warrant sex-stratified analyses in future interventional studies [29,65,67,68,69].

A significant finding is that the relationships between B12 and lipid parameters are generally consistent across age groups (18–44, 45–59, 60–74, 75+). B12 × age group interactions were not found to be significant for AIP, triglycerides, HDL-C, and VLDL-TG, suggesting that the effects of B12 on lipid metabolism are age-independent. Given the higher prevalence of B12 deficiency in the elderly population, B12 screening and, if necessary, replacement for this group could be considered as part of cardiovascular protection strategies. B12 deficiency has been reported to reach 10–15% in the elderly [64].

Our findings have several important clinical implications. Firstly, they suggest that B12 status could be used as a potential biomarker in cardiovascular risk assessment. In particular, its strong correlation with AIP could be helpful in identifying individuals at risk of subclinical atherosclerosis. Current American Heart Association guidelines do not recommend B12 measurement in cardiovascular risk assessment [70]; however, our findings point to the potential benefit of B12 assessment, particularly in individuals with metabolic risk factors. Secondly, whether correcting B12 deficiency has positive effects on lipid profiles in dyslipidemic patients is a subject worth investigating. In a study conducted in Türkiye [60], significant reductions in serum cholesterol (from 209.6 ± 30.1 mg/dL before treatment to 195.6 ± 31.7 mg/dL after treatment) and triglyceride levels (from 196.2 ± 117.8 to 142.1 ± 81.4 mg/dL) were reported in 21 patients receiving B12 treatment. A decrease in triglyceride levels was observed in all patients. While these findings are promising, they need to be confirmed by randomized controlled trials due to the small sample size and the lack of a control group. Building upon these general clinical implications, the age and sex-stratified analyses provide important insights for clinical practice. The pronounced B12-cholesterol relationship in middle-aged adults suggests that routine B12 screening should be prioritized in individuals aged 45–74 years, particularly those with dyslipidemia or multiple cardiovascular risk factors. The timing of screening may be critical, as intervening during this window could prevent or attenuate age-related deterioration in lipid profiles. Furthermore, the substantial sex differences indicate that risk stratification algorithms should incorporate sex-specific B12 thresholds. While women appear to maintain favorable lipid profiles even at marginal B12 levels (148–221 pg/mL), men in this category may already exhibit intermediate cardiovascular risk, warranting earlier supplementation. This is particularly relevant for men over 50 years with additional risk factors such as obesity, sedentary lifestyle, or family history of cardiovascular disease, who may benefit from maintaining B12 levels in the upper quartile (>540 pg/mL) to optimize lipid parameters. From a broader clinical perspective, the integration of B12 measurement into the routine assessment of patients with dyslipidemia deserves consideration. In daily clinical practice, dyslipidemia management primarily focuses on statin therapy, lifestyle modifications, and monitoring of traditional lipid parameters [41,49]. However, our findings suggest that B12 status may represent an overlooked modifiable factor that contributes to the atherogenic lipid phenotype, particularly through its association with triglyceride-rich lipoproteins and AIP. This may be especially relevant in high-risk populations, including patients with type 2 diabetes on metformin therapy, elderly individuals with malabsorption, and patients with chronic kidney disease or metabolic syndrome. A recent Delphi consensus on B12 deficiency management has emphasized the importance of timely screening in at-risk groups [9,71]. In the context of dyslipidemia, incorporating B12 assessment alongside conventional lipid panels could identify patients in whom B12 optimization might complement standard lipid-lowering strategies. Although causality has not been established, the consistent association between higher B12 levels and a more favorable lipid profile across our large cohort suggests that prospective trials evaluating B12 supplementation as an adjunct to dyslipidemia management are warranted, particularly in populations with documented B12 deficiency.

### Strengths and Limitations

The strengths of our study include a large sample size (*n* = 20,665), detailed lipoprotein subfraction analysis (VLDL1-TG, VLDL5-TG, LDL1-TG, LDL2-TG, LDL-5-TG, LDL-6-TG), stratified assessment by age groups, and multiple comparison corrections (Tukey HSD and Bonferroni). While most studies in the literature assess total VLDL or LDL levels, our study presents analysis at the level of specific subfractions. Furthermore, the reporting of effect sizes (partial η^2^) allows for the evaluation of the clinical significance of the findings. However, several limitations exist. Due to the cross-sectional design, causality cannot be established; it is unclear whether the observed associations reflect a direct effect of B12 or concomitant factors. Importantly, potentially relevant confounders, including body mass index (BMI), dietary intake, alcohol consumption, smoking status, physical activity level, renal function, glucose metabolism, and medication use (especially metformin and proton pump inhibitors), were not available in the dataset and could not be included in multivariable adjustment. Given that the effect size of sex on AIP (partial η^2^ = 0.142) was substantially larger than that of B12 (partial η^2^ = 0.017), the observed B12–lipid associations may be attenuated or modified after adjustment for these additional confounders. Future studies employing fully adjusted multivariable regression models are warranted to disentangle the independent contribution of B12 from other cardiometabolic determinants. A key methodological limitation is that serum B12 alone is an imperfect biomarker of functional B12 status. As highlighted by Herrmann et al. [17], serum B12 levels may not fully reflect cellular B12 status; assessment of functional markers such as holotranscobalamin or methylmalonic acid could provide a more comprehensive picture. The absence of these functional biomarkers limits our ability to distinguish between true cellular B12 deficiency and low serum levels that may not reflect intracellular status. Future studies should incorporate these markers to improve biological interpretation. Several suggestions can be offered for future research in this area. Longitudinal cohort studies could assess the impact of changes in B12 status on lipid profile and cardiovascular events. Randomized controlled trials could test the effect of B12 supplementation on lipid parameters in dyslipidemic patients, particularly in populations without folate fortification, as in the Chinese CSPPT study [72]. Mechanistic studies could elucidate the effects of B12 on hepatic lipid metabolism, lipoprotein lipase activity, CETP function, and HDL functionality at the molecular level.

## 5. Conclusions

In conclusion, this large-scale cross-sectional study reveals that serum vitamin B12 levels are significantly associated with atherogenic lipid profiles. Higher B12 concentrations are consistently associated with lower AIP, reduced triglyceride load, improved HDL-cholesterol levels, and lower VLDL-TG fractions. Notably, the B12-associated decrease in LDL5-TG levels, the most atherogenic LDL triglyceride subfraction, was consistently observed across all age groups, reinforcing the potential relevance of B12 status in cardiovascular risk assessment. These findings are largely consistent with previous clinical and experimental studies [1,2,14,30,41]. The strong effect on AIP, combined with the consistency across age groups, indicates that B12 status could serve as a valuable marker in cardiovascular risk stratification. In clinical practice, assessment and optimization of B12 status, especially in individuals with cardiovascular risk factors, should be considered as part of a comprehensive cardiovascular protection strategy. However, it should be noted that serum B12 levels may not fully reflect functional B12 status, and the absence of holotranscobalamin or methylmalonic acid measurements limits biological interpretation. Prospective longitudinal studies incorporating functional B12 biomarkers and randomized controlled trials are needed to establish causality and to evaluate the effect of B12 intervention on cardiovascular outcomes.

## Figures and Tables

**Table 1:** Basic demographic and lipid/lipoprotein characteristics of participants according to serum vitamin B12 quartiles.

Characteristics	Total (*n* = 20,665)	Q1 (*n* = 5175) ≤328 pg/mL	Q2 (*n* = 5204) 329–417 pg/mL	Q3 (*n* = 5131) 418–539 pg/mL	Q4 (*n* = 5155) ≥ 540 pg/mL	*p* Value
Age (years)	45.85 ± 13.459	45.77 ± 13.173	44.79 ± 12.880	45.50 ± 13.197	47.37 ± 14.412	<0.001
**Sex, *n* (%)**						
*Female*	9193 (%44.5)	2163 (%41.8)	2240 (%43.0)	2233 (%43.5)	2557 (%49.6)	<0.001
*Male*	11,472 (%55.5)	3012 (%58.2)	2964 (%57.0)	2898 (%56.5)	2598 (%50.4)	
Vitamin B12 (pg/mL)	461 ± 207.09	268.71 ± 44.44	372.22 ± 25.34	472.32 ± 34.60	732.34 ± 221.99	<0.001
TG (mg/dL)	120.76 ± 91.27	132 ± 100	124.75 ± 97.78	117.62 ± 85	108.58 ± 78.32	<0.001
HDL-C (mg/dL)	58.52 ± 14.28	55.65 ± 13.33	57.84 ± 13.91	59.07 ± 14.42	61.48 ± 14.79	<0.001
LDL-C (mg/dL)	130.89 ± 38.91	127.77 ± 38.10	130.25 ± 37.43	131.90 ± 39.15	133.66 ± 40.69	<0.001
Total cholesterol (mg/dL)	216.62 ± 47.11	213.85 ± 46.87	216.43 ± 46.02	217.49 ± 46.91	218.74 ± 48.50	<0.001
AIP	−0.107 ± 0.303	−0.050 ± 0.305	−0.091 ± 0.303	−0.119 ± 0.299	−0.107 ± 0.294	<0.001
Total VLDL-TG (mg/dL)	75.37 ± 69.13	84.13 ± 75.16	78.45 ± 73.59	73.28 ± 64.97	65.55 ± 60.30	<0.001
Total LDL-TG (mg/dL)	19.76 ± 7.74	19.96 ± 7.87	19.65 ± 7.67	19.52 ± 7.31	19.88 ± 8.09	=0.014

Data are presented as mean ± SD or *n* (%). Interquartile comparisons for continuous variables were performed using one-way ANOVA, and for categorical variables (sex), the chi-square (χ^2^) test was used. AIP was calculated as log_10_(TG/HDL-C). *p* < 0.05 was considered statistically significant.

**Table 2:** Atherogenic Index of Plasma (AIP) values stratified by vitamin B12 quartiles and sex.

B12 Quartile (pg/mL)	Female (*n* = 9193)	Male (*n* = 11,472)	Total (*n* = 20,665)
Q1 (≤328)	−0.184 ± 0.266	0.045 ± 0.295	−0.051 ± 0.305
Q2 (329–417)	−0.231 ± 0.258	0.014 ± 0.293	−0.091 ± 0.304
Q3 (418–539)	−0.253 ± 0.250	−0.016 ± 0.294	−0.119 ± 0.299
Q4 (540+)	−0.273 ± 0.250	−0.016 ± 0.294	−0.170 ± 0.294
Total	−0.237 ± 0.258	−0.005 ± 0.298	−0.108 ± 0.304
*p* (B12 effect)	<0.001	<0.001	<0.001

Data are presented as mean ± standard deviation. AIP was calculated using the formula log_10_(TG/HDL-C). AIP risk categories: <0.11 (low risk), 0.11–0.24 (medium risk), >0.24 (high risk). Two-way ANOVA analysis was performed for the quartile effect of B12 within each sex. Two-way ANOVA results: B12 quartile effect (F = 120.589; *p* < 0.001; partial η^2^ = 0.017), sex effect (F = 3423.521; *p* < 0.001; partial η^2^ = 0.142), B12 × sex interaction (F = 5.345; *p* = 0.001; partial η^2^ = 0.001).

## Data Availability

The data supporting the findings of this study are available from the corresponding author upon reasonable request. The datasets are not publicly available due to institutional data management policies, ethical considerations, and confidentiality requirements.

## Supplementary Materials

The following supporting information can be downloaded at: https://www.mdpi.com/article/10.3390/jcm15051775/s1, Figure S1: Sex-specific distribution of serum Vitamin B12 levels; Figure S2: Total Cholesterol Distribution Across Vitamin B12 Quartiles Stratified by Age Groups; Figure S3: Age-Stratified Analysis of VLDL-TG Subfractions Across Vitamin B12 Quartiles; Figure S4: Effect Size Analysis of Vitamin B12 Associations with VLDL-TG Subfractions.

## Abbreviations

The following abbreviations are used in this manuscript:

NMR	Nuclear Magnetic Resonance
AIP	Atherogenic Index of Plasma
CVD	Cardiovascular Diseases
HDL	High-Density Lipoprotein
LDL	Low-Density Lipoprotein
VLDL	Very Low-Density Lipoprotein
TG	Triglycerides
sdLDL	Small Dense Low Density Lipoprotein
T2DM	Type 2 Diabetes Mellitus
tHcy	Total Homocysteine
TSP	3-(trimethylsilyl) propionic-2,2,3,3-d4 acid
GLM	General Linear Model
