# Supplementary material for: Vitamin B12 Status and Cardiovascular Risk: Novel Insights from NMR-Based Lipoprotein Profiling in 20,665 Adults
Source: J Clin Med. 2026 Feb 26;15(5):1775. doi: 10.3390/jcm15051775 (PMC12985633; doi:10.3390/jcm15051775)
Supplement: Supplementary file 1 [file jcm-15-01775-s001.zip › jcm-4162421-supplementary.pdf]

Supplementary Materials:

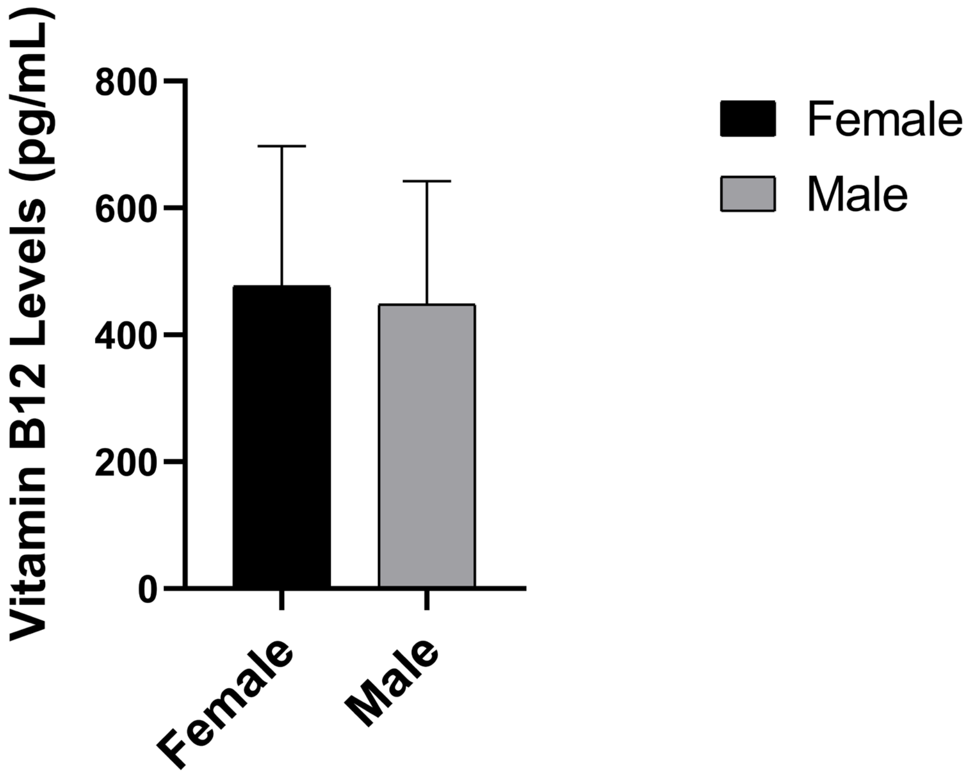

Supplementary Figure S1. Sex-specific distribution of serum Vitamin B12 levels. Serum Vitamin B12 concentrations (pg/mL) compared between female and male participants. Data are presented as mean ± SD for female (n = 9,193) and male (n = 11,472) subjects. Significant difference was observed between groups ($p < 0.001$).

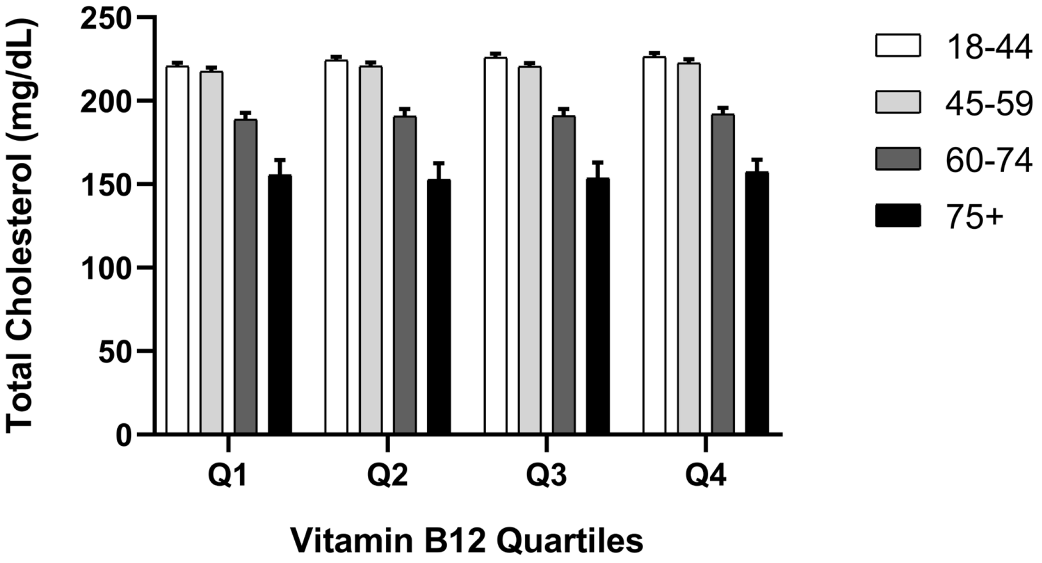

Supplementary Figure S2. Total Cholesterol Distribution Across Vitamin B12 Quartiles Stratified by Age Groups. Total cholesterol levels (mg/dL) across vitamin B12 quartiles (Q1: ≤328 pg/mL; Q2: 329-408 pg/mL; Q3: 409-540 pg/mL; Q4: >540 pg/mL) stratified by age categories. Data presented as mean with 95% confidence intervals (CI). An inverse relationship between B12 status and total cholesterol was observed across all age groups, with the most pronounced differences in middle-aged adults (45-59 and 60-74 years). Higher B12 quartiles were associated with lower total cholesterol levels, particularly in the 45-59 age group where Q1 to Q4 difference was approximately 18 mg/dL. Statistical analysis: Two-way ANOVA with age as covariate. Error bars represent 95% CI.

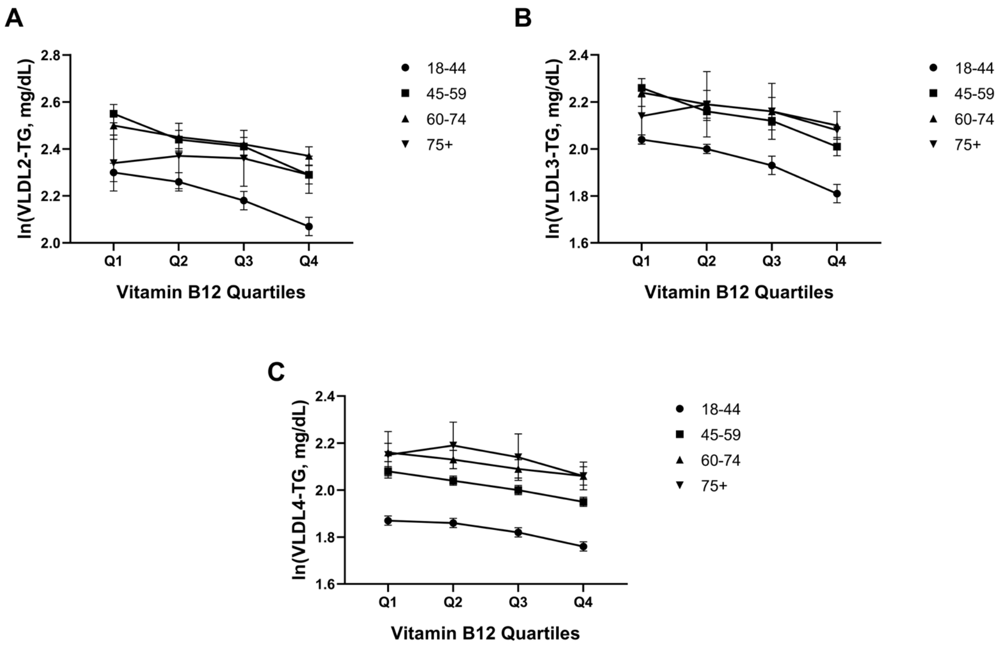

Supplementary Figure S3. Age-Stratified Analysis of VLDL-TG Subfractions Across Vitamin B12 Quartiles. Natural log-transformed VLDL-TG subfractions stratified by age groups across vitamin B12 quartiles (Q1: ≤328 pg/mL; Q2: 329-408 pg/mL; Q3: 409-540 pg/mL; Q4: >540 pg/mL). (A) ln(VLDL2-TG) - intermediate-sized particles;(B): ln(VLDL3-TG) - smaller particles; (C): ln(VLDL4-TG) - small particles. Age groups: 18-44, 45-59, 60-74, and 75+ years. Data represent estimated marginal means with 95% confidence intervals (CI) from generalized linear model (GLM) analysis. Error bars represent 95% CI. Consistent inverse relationships between B12 status and VLDL-TG levels were observed across all age groups ($p<0.001$). While baseline VLDL-TG increased with age (particularly in 45-74 years), the protective association of higher B12 quartiles was maintained across the lifespan. The most substantial differences between B12 quartiles occurred in middle-aged adults (45-74 years), indicating that adequate B12 status may be particularly critical for cardiovascular risk management during this period. Even in adults aged 75+ years, higher B12 quartiles were associated with lower VLDL-TG levels.

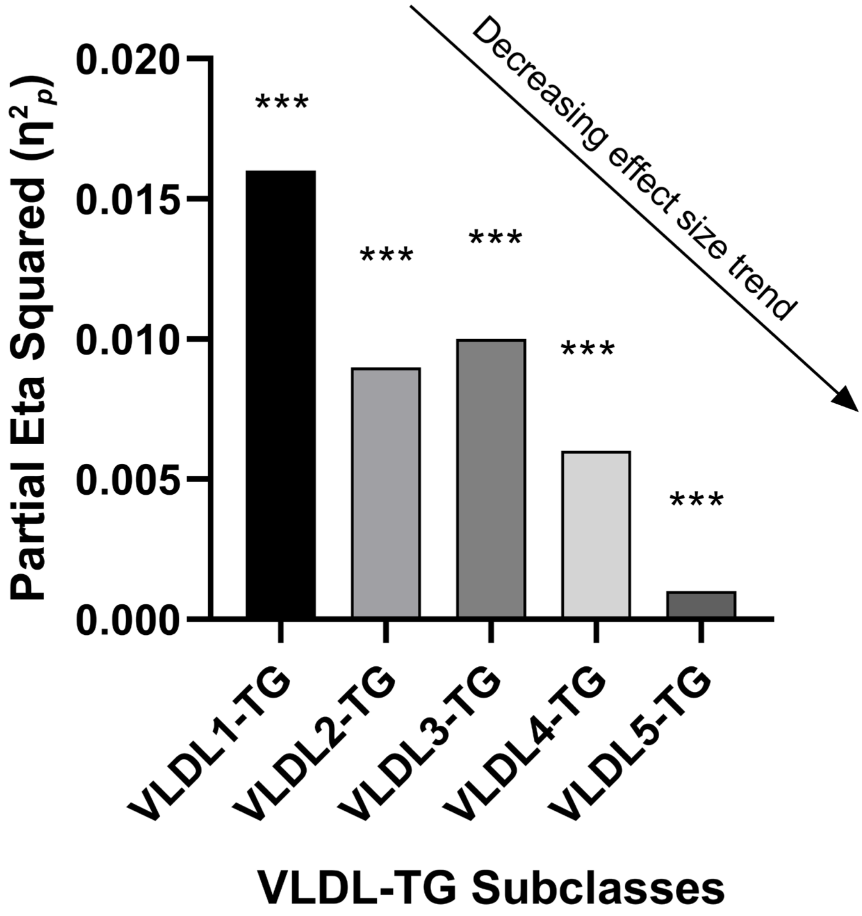

Supplementary Figure S4. Effect Size Analysis of Vitamin B12 Associations with VLDL-TG Subfractions. Partial eta squared (η^2_p) values demonstrating the strength of associations between vitamin B12 quartiles and VLDL-TG subfractions (VLDL1-TG through VLDL5-TG). Effect sizes were calculated using generalized linear model (GLM) analysis adjusted for age. All associations were statistically significant (***$p<0.001$). The decreasing trend from VLDL1-TG to VLDL5-TG indicates that the association between B12 and VLDL-TG is strongest for larger, hepatically secreted VLDL particles rather than smaller remnant particles. VLDL1-TG showed the largest effect size (η^2_p = 0.016), followed by VLDL3-TG (η^2_p = 0.010), VLDL2-TG (η^2_p = 0.009), VLDL4-TG (η^2_p = 0.006), and VLDL5-TG (η^2_p = 0.001).
